# Mechanism of opioid addiction and its intervention therapy: Focusing on the reward circuitry and mu‐opioid receptor

**DOI:** 10.1002/mco2.148

**Published:** 2022-06-22

**Authors:** Jia‐Jia Zhang, Chang‐Geng Song, Ji‐Min Dai, Ling Li, Xiang‐Min Yang, Zhi‐Nan Chen

**Affiliations:** ^1^ National Translational Science Center for Molecular Medicine & Department of Cell Biology The Fourth Military Medical University Xi'an China; ^2^ Department of Neurology Xijing Hospital The Fourth Military Medical University Xi'an China; ^3^ Department of Hepatobiliary Surgery Xijing Hospital The Fourth Military Medical University Xi'an China

**Keywords:** dependence, mu‐opioid receptor, opioid addiction, reward circuitry, withdrawal syndrome

## Abstract

Opioid abuse and addiction have become a global pandemic, posing tremendous health and social burdens. The rewarding effects and the occurrence of withdrawal symptoms are the two mainstays of opioid addiction. Mu‐opioid receptors (MORs), a member of opioid receptors, play important roles in opioid addiction, mediating both the rewarding effects of opioids and opioid withdrawal syndrome (OWS). The underlying mechanism of MOR‐mediated opioid rewarding effects and withdrawal syndrome is of vital importance to understand the nature of opioid addiction and also provides theoretical basis for targeting MORs to treat drug addiction. In this review, we first briefly introduce the basic concepts of MORs, including their structure, distribution in the nervous system, endogenous ligands, and functional characteristics. We focused on the brain circuitry and molecular mechanism of MORs‐mediated opioid reward and withdrawal. The neuroanatomical and functional elements of the neural circuitry of the reward system underlying opioid addiction were thoroughly discussed, and the roles of MOR within the reward circuitry were also elaborated. Furthermore, we interrogated the roles of MORs in OWS, along with the structural basis and molecular adaptions of MORs‐mediated withdrawal syndrome. Finally, current treatment strategies for opioid addiction targeting MORs were also presented.

## Graphical abstract

1. Rewarding effects and withdrawal syndrome are two dominant components of opioid addiction.

2. Mu‐opioid receptors (MORs) are highly involved in reward circuitry and the development of withdrawal syndrome.

3. MORs are important targets to treat addiction and withdrawal syndrome.

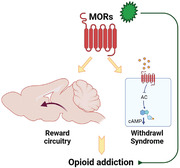

## 1 INTRODUCTION

Opioids have been applied for thousands of years in human history to relieve pain. The medicinal application of opioids can be dated back to 1500 B.C. when people used opioids to treat “excessive crying of baby.”^1^ Over the last decades, opioids have long been used as the most powerful analgesics and remain the most frequently used analgesics against severe pain.^2^, ^3^ Currently, opioids are widely used in acute pain^4^, ^5^ and cancer pain,^6^, ^7^, ^8^, ^9^ and are especially noticed and prescribed in chronic pain.^10^, ^11^, ^12^, ^13^, ^14^, ^15^, ^16^, ^17^, ^18^ Apart from their analgesic effects, opioids, both prescribed opioid analgesics (morphine, hydrocodone, oxycodone hydrochloride) and illicit opioids (heroin and its analogs) are associated with the propensity for addiction.^19^ Drug addiction is characterized by a recurring desire to continue taking the drug despite harmful consequences.^20^, ^21^, ^22^ Five elements of addiction have been identified from the literature, including (1) engaging processes to achieve appetitive effects, (2) preoccupation with the behavior, (3) temporary satiation, (4) loss of control, and (5) harmful consequences.^23^

Opioid addiction has become an epidemic and global concern in recent years. It was estimated that 26.8 million people had opioid use disorder globally, with over 100,000 opioid overdose deaths annually.^24^, ^25^ The prevalence of opioid use disorder is highest in the United States.^25^ It was estimated that over 4% of the adult population (more than 10 million Americans) currently misuse prescription opioids.^26^ There was an average of five Americans per hour who died from opioid overdose.^27^ Moreover, the problem of opioid addiction is even more complicated by the intractable dependence on opioids and the high likelihood of relapse.^28^ Tremendous socioeconomic burden, along with the impact on health and well‐being, has been cast on both society and addicted individuals.^29^, ^30^, ^31^ The higher economic cost could be attributed to health care and substance abuse treatment costs, workplace costs due to lost earnings and lost employment, and criminal justice costs.^29^, ^32^ Moreover, drug addiction and substance abuse have already shed shadows on young people, poisoning their mental and physical health and hindering their social development.^33^, ^34^ Thus, it is of vital importance to understand the mechanism of opioid addiction and urgent to treat opioid addiction based on related mechanism.

Exogenous and endogenous opioids exert their biological effects via opioid receptors, which belong to the superfamily of seven transmembrane (TM) G‐protein coupled receptors (GPCRs).^35^, ^36^, ^37^, ^38^, ^39^ Mu‐opioid receptors (MORs), a member of opioid receptors, are dominant in mediating both the analgesic and addictive effects of opioids.^40^, ^41^, ^42^, ^43^, ^44^, ^45^ Opioid addiction is a complex process in which compulsive seeking for rewarding and euphoric effects is initially involved and succeeded by dependence on opioids, which usually leads to the failure of the attempt to quit drug abuse and reinforcement of addiction.^27^, ^46^ Noticeably, the target of opioids, MORs, play important roles in both the regulation of neural circuitry of the reward system and the cellular adaptions of chronic opioids exposure that cause dependence and withdrawal syndromes.^43^, ^47^, ^48^, ^49^, ^50^

Currently, most therapeutic strategies for opioid addiction target MORs, which are also the targets for drug development.^51^ Thus, considering the central regulatory role of MORs in both reward and dependence aspects of drug addiction, fully understanding the mechanism of MORs in opioid addiction is the cornerstone of effective management of opioid abuse disorders and opioid addiction. In this review, we discuss the structural and functional characteristics of MORs that may have implications for opioid addiction. We thoroughly summarize the circuitry of the brain reward system and the progress in the understanding of the role of MORs within the reward circuitry, as well as the roles of MORs in the development of opioid withdrawal symptoms. Finally, we briefly review current treatment strategies for opioid addiction targeting MORs.

## 2 MORs

Since ancient times, opioids have been used for their analgesic and psychotropic effects. Despite the therapeutic effects, opioid drugs are also associated with undesired effects, such as addiction (for the definition of addiction, please refer to the Introduction section of this review), dependence (in this review, dependence refers to physical dependence, which manifests as the emergence of withdrawal symptoms when the repeated use of opioids is abruptly stopped or tapered and often pushes users to seek opioids to avoid withdrawal syndrome, resulting in the relapse of opioid misuse and reinforced addiction),^19^ tolerance (meaning that an increased dose of opioids is needed to achieve the same therapeutic effects, especially when opioids are taken as analgesics),^52^ and abstinence reactions (abstinence means abstaining from the abused drugs, and abstinence reactions here are equal to withdrawal reactions or withdrawal syndrome characterized by symptoms such as muscle and joint pain, diarrhea, cramps, nausea, vomiting, runny nose, insomnia, dysphoria, anxiety, and irritability).^53^ For a more detailed review of withdrawal syndrome, refer to Section 4). Both therapeutic and unwanted effects of opioid drugs were exerted through their binding to MORs. In mice with MOR deletion, the analgesic effect of morphine along with its dependence and rewarding effects are simultaneously abolished.^54^ Thus, MORs are regarded as the molecular target for opioids such as morphine, fentanyl, methadone, and the notorious heroin that is widely used today.

### 2.1 Structure of MORs

Classical opioid receptors mainly include three subtypes, the MORs, the delta‐opioid receptors (DORs), and the kappa‐opioid receptors (KORs), which are encoded by *OPRM1*, *OPRD1*, and *OPRK1*, respectively.^43^ The discovery of multiple receptors, MORs, DORs, and KORs came from the demonstration of different profiles of pharmacological activity with the prototype agonists morphine, ketazocine, and N‐allylnormetazocine. These receptors belong to the inhibitory GPCR superfamily, which mediates inhibitory signaling upon activation. The typical protein structure of these receptors is characterized by seven TM domains, an extracellular N‐terminus, and an intracellular C‐terminus inside the cells.^55^ More recently, cDNA encoding an “orphan” receptor was also identified, which has a high degree of homology to the “classical” opioid receptors. The m1/m2 subdivision of MORs was proposed by Pasternak and colleagues to explain their observations that [^3^H]‐labeled MORs ligand displayed biphasic binding characteristics.^56^ Naloxazone and naloxonazine were reported to abolish the binding of radioligand to the m1‐site^57^ (ref: https://www.opioids.com/receptors/index.html)

Alternative splicing is another prominent feature of MORs in cells. Alternative splicing is a regulatory mechanism of gene expression that allows the generation of multiple mRNA species from an individual gene.^58^ Beyond the seven highly homogeneous TM regions, divergent alternative splicing occurs at the upstream and downstream terminals of MORs mRNA.^59^ Till now, there are 19 transcript variants that have been listed in the Aceview database of NCBI of homo species (UCSC genome browser GRCh37/hg19; for more detailed information, refer to the review by Pasternak and Pan^55^). In the rat central nervous system (CNS), MOR1, MOR1A, and MOR1B can be detected, and the mRNA levels of MOR1 and MOR1A are significantly higher than those of MOR1B.^60^ Moreover, in HEK293 cells expressing these receptors, [D‐Ala2, N‐Me‐Phe4, ‐Gly‐ol5] encephalin (DAMGO)‐induced desensitization was significantly lower for MOR1B than for MOR1 and MOR1A.^60^ C‐terminal splicing of MORs might modulate agonist‐induced internalization and re‐sensitization of MORs. The DAMGO‐induced internalization of MOR1B proceeds even faster effect than that of MOR1, followed by rapid recycling of the detected receptor to the cellular surface.^61^ After opioid removal, functional recovery (re‐sensitization) of MOR1B was also significantly impaired when compared with MOR1.^62^ Other reports also showed that MORs splicing variants are associated with divergent roles for the C‐terminal in morphine‐induced behaviors. In mice with selectively truncated C‐terminal tails encoded by exon 7 transcript variant, morphine‐induced reward and tolerance effects are diminished without notably altering physical dependence, whereas in mice selectively expressing truncated C‐terminal tails encoded by exon 4 transcript variant, morphine tolerance is facilitated, and morphine dependence is reduced without interfering with morphine reward.^63^ Interestingly, the effect of morphine on different MORs splicing variant seems to be sex‐dependent. Chronic systemic morphine results in a twofold increase in the levels of spinal cord MOR1B2 and MOR1C1 in male rats, but this effect is completely absent in females,^64^ indicating the importance of sex‐specific mechanisms of morphine tolerance and addiction in vivo.

The crystal structure of MOR has been reported by Manglik et al.^65^ By using the T4 lysosome fusion protein strategy, the authors obtained the crystal structure of the complex in which MORs bind with the irreversible morphinan antagonist β‐funaltrexamine (β‐FNA). The MORs’ structure generally consists of seven TM alpha‐helices, among which the alpha‐helices are connected by three extracellular loops (ECLs 1–3) and along with three intracellular loops. Also, TM3 is connected to ECL2 by a conserved disulfide bridge bond between C140 and C217. Notably, unlike most GPCR, where the ligand‐binding pocket is buried within the helical bundle by superficial residues in TMs and ECL2, such as M2 and M3 muscarinic acetylcholine receptors,^66^, ^67^ the binding pocket of MORs for β‐FNA is largely exposed to the portion of the extracellular surface. This observed ligand‐binding pocket may partially explain the rapid dissociation half‐lives for potent opioids such as alvimopan, etorphine, and diprenorphine.^68^, ^69^ Moreover, MORs were observed to readily dimerize and form oligomers. The homogeneous dimers of MORs are formed through the interface of 28 residues in the structure of TM5 and TM6, and oligomers of MORs dimers are formed through the parallel association mediated by the structure of TM1, TM2, and helix eight; however, the function of the oligomers is still poorly understood.

### 2.2 Distribution of MORs

MORs are widely distributed in the CNS and peripheral nervous system. In the CNS, MORs can be detected in a variety of brain regions, including the neocortex, hippocampus, striatum, amygdala, thalamus, hypothalamus, periaqueductal gray (PAG), medulla, and pons, where they exert a certain function in local circuits. MORs are suggested to be located in the cerebral neocortex. In the cerebral neocortex, opioid was reported to release at the orbitofrontal cortex after alcohol exposure, and the changes in the opioid of the orbitofrontal cortex correlated significantly with the alcohol‐use behavior.^70^ Moreover, in the insular cortex, MORs play an important role to mediate long‐term synaptic depression at the inputs to the dorsolateral (DL) striatum.^71^ In the prefrontal cortex (PFC), MORs are also engaged in the regulation of network that controls appetitively motivated behaviors, and the disruption of such network is associated with impulsive appetitive response.^72^ In the hippocampus, MORs were mainly found in GABAergic inhibitory interneurons, including parvalbumin (PV)‐expressing basket cells, neuropeptide Y‐expressing interneurons, vasoactive intestinal peptide‐expressing interneurons, somatostatin‐expressing interneurons, and calretinin‐containing interneurons.^73^ In addition, in the hippocampus, MORs were also detected in ivy and neurogliaform interneurons.^74^ Hippocampal MORs were also reported to be involved in the modulation of sharp waves and ripples and thus the hippocampus‐dependent memory.^75^ In the CA3 region of the hippocampus, MORs play a vital role in the acquisition and retrieval of spatial memory.^76^ In the striatum, MORs were reported to be expressed by striatal projection neurons in both the matrix and the patches, the two distinct structural divisions of the striatum, and also in cholinergic interneurons.^77^, ^78^ Selective activation of MORs in cholinergic interneurons in the dorsal lateral striatum caused a strong inhibition of firing activity in the cholinergic interneurons, which was believed to underlie the pathogenesis of dystonia.^78^ Striatal MORs have also been shown to contribute to methamphetamine‐induced stereotypy^79^ and opioid‐induced locomotor sensitization,^80^ suggesting a role of striatal MORs in drug effects. In amygdala intercalated neurons, MORs play a vital role in processing the information between the basolateral complex of the amygdala and central nuclei of the amygdala.^81^ The amygdala MOR system has also been declared to regulate reward behavior and appetite behavior.^82^

MORs were also reported to be located in major nuclei of the thalamus and hypothalamus. Thalamic MORs are involved in the modulation of pain esthesia.^83^, ^84^, ^85^, ^86^ MORs in the parafascicular nucleus of the thalamus were reported to mediate the effects of morphine‐induced antinociception.^87^ Hypothalamic MORs, interestingly, are involved in the regulation of the hypothalamic–pituitary–adrenal axis, as evidenced by the fact that the A118G polymorphism of MORs blunted the adrenocorticotropic hormone response to metyrapone.^88^ Notably, hypothalamic MORs mediated the effects of drug abuse. MORs were reported to mediate the depression of the hypothalamic hypocretin/orexin arousal system, which may explain the sedation and mental lethargy after morphine exposure.^89^ Moreover, MORs on the microglia in the hypothalamus contributed to the neuroinflammation process after alcohol exposure, although glial MORs are not within the central topic of the current review.^90^

In more posterior regions like PAG, medulla, and pons, MORs also exist and exert functions. PAG is a midbrain region that is involved in the modulation of nociception, and MORs in the PAG are important targets for analgesia.^91^, ^92^, ^93^ It was reported that the activation of MORs in the ventral PAG could decrease the inhibitory inputs onto the dopamine (DA) neurons in the ventral PAG, the activation of which displayed an antinociceptive effect.^91^ Recently, Kandasamy et al. found that positive allosteric modulators targeting MORs in the PAG produced antinociception with reduced levels of morphine‐induced side effects such as reward and respiratory depression.^94^ This study demonstrated that positive allosteric modulators of MORs, rather than traditional opioid drugs such as morphine, might be more suitable analgesics with few side effects. MORs in the medulla had two important aspects associated with unwanted effects of morphine‐induced analgesia. For one thing, considering medulla is the home of respiratory centers, MORs activation in the medulla inhibited the respiratory centers, thus mediating the side effect of respiratory depression of morphine.^95^ In addition, it was reported that the complex formed by vasopressin 1b receptor, β‐arrestin‐2, and MORs in the rostral ventromedial (VM) medulla mediated morphine tolerance.^96^ Similar to MORs in the medulla, MORs in the pons mediated the respiratory depression of morphine. The pre‐Bötzinger complex, a respiratory rhythm‐generating area in the pons, is inhibited upon MORs activation,^97^ while the pontine respiratory‐controlling Kölliker‐Fuse neurons, which maintain upper airway patency and a normal respiratory pattern, could be hyperpolarized by MORs, leading to the suppression of post‐inspiratory drive.^98^

As for subcellular location, MORs localize in different parts of the neuron, that is, axonal terminals, dendrites and soma, and exhibit distinct functional properties. First, activation of differentially distributed MORs could exert different electrophysiological effects. The activation of MORs in the somatodendric compartment decreases cellular excitability, whereas the activation of MORs in the axonal terminal inhibits neurotransmitter release, resulting in decreased downstream excitation or disinhibition.^99^ Second, agonist‐induced MORs desensitization differs between MORs located in the nerve terminals and those in the cell bodies. The high‐efficacy MORs agonist DAMGO, an MOR selective agonist with a Kd of 3.46 nM for native MORs, could induce rapid MOR desensitization at the ventral tegmental area (VTA)

GABAergic neuron bodies but not at the terminals. However, after prolonged treatment (> 7 h) with Met‐enkephalin, one of endogenous opioid peptides isolated from the porcine brain in 1975, both MORs in the terminals and cell bodies exhibit profound desensitization.^100^

In the peripheral nervous system, MORs are mainly implicated in nociception. An elucidated description of the detailed role of peripheral MORs can be found in the review by Rauck.^101^

### 2.3 Endogenous MOR ligands

Several endogenous MOR ligands have been reported, including β‐endorphin, Met‐enkephalin, Leu‐enkephalin, and so forth. These endogenous MOR ligands generally contain a Tyr‐Gly‐Gly‐Phe‐Met/Leu sequence at the N‐terminals, which can be recognized as the opioid motif.^40^ Another kind of endogenous opioid receptor ligand, endomorphin‐1/2, is characterized by the opioid motif being substituted with peptides Tyr‐Pro‐Trp‐Phe‐NH2 and Tyr‐Pro‐Phe‐Phe‐NH2, and they showed high selectivity and affinity for MORs.^102^ Upon binding to MORs, endogenous opioid ligands, that is, β‐endorphin, enkephalin, and endomorphin, can exert distinct functions promptly. Intra‐nucleus accumbens (NAc) administration of β‐endorphin could increase social play behavior, a highly rewarding social interaction in adolescent rodent species, but the administration of met‐enkephalin could not induce similar effects.^103^ In the arcuate nucleus of the hypothalamus, β‐endorphin plays an important role in antinociception behavior.^104^ In addition, a compensatory increase in enkephalin release during morphine withdrawal could promote a second period of MOR activity, which is responsible for the enhanced naloxone (NX) aversion.^105^ Moreover, in the pre‐Bötzinger complex, which is the center of respiratory rhythm generation, endomorphin‐2 could play a promotive role in respiratory depression through MORs.^106^ Endogenous opioid peptides can be inactivated by aminopeptidase N and enkephalinases.^40^ Inhibition of the degradation of endogenous opioid peptides in both peripheral injured tissues and the CNS could produce analgesic effects.^107^, ^108^

### 2.4 Biased signaling of MORs

**FIGURE 1:**
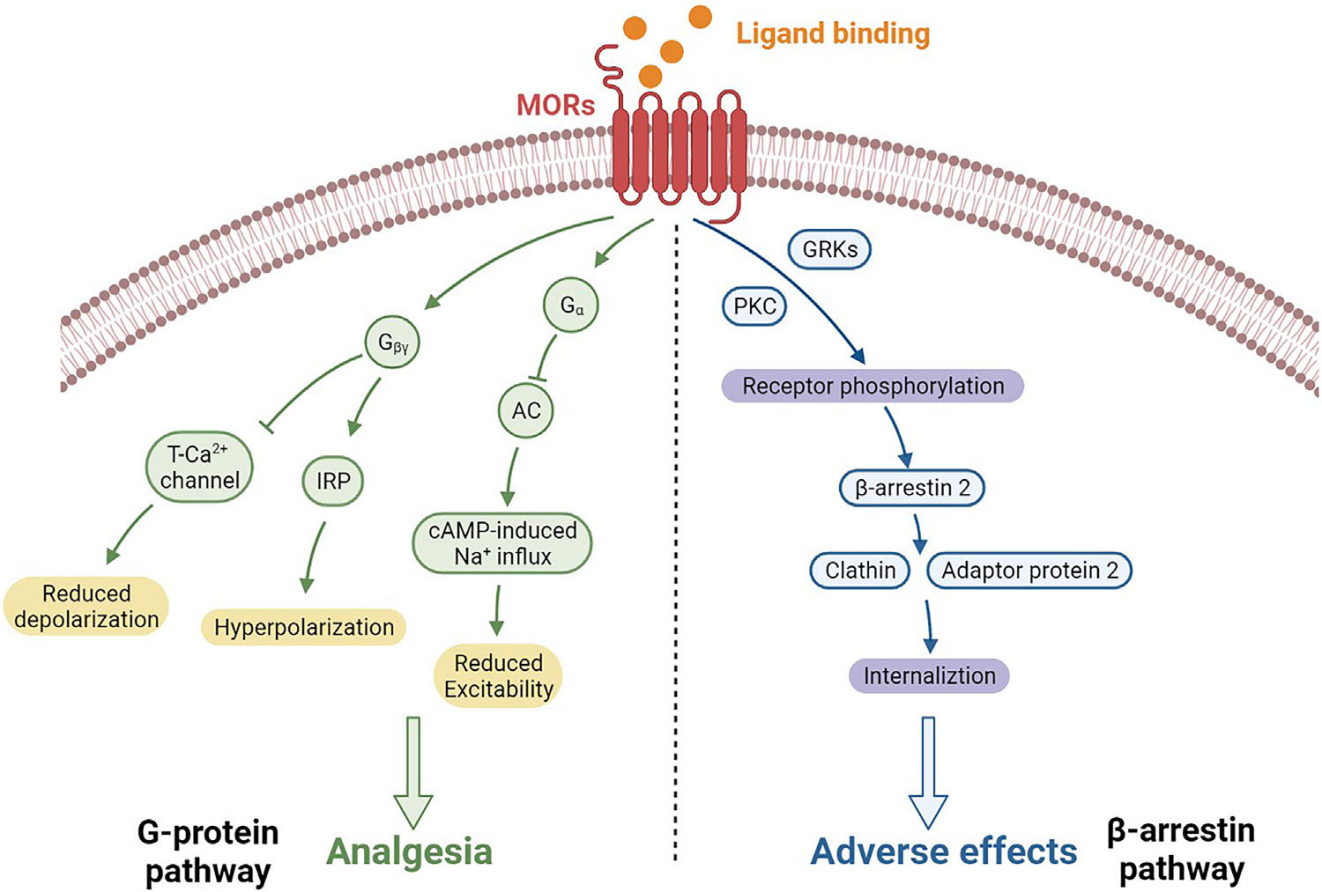
Biased signaling of Mu opioid receptors (MORs). The analgesic effects and adverse effects of MORs ligands are mediated by the G‐protein pathway and β‐arrestin pathway, respectively. The analgesic effects are mediated by G proteins, which inhibit AC, activate IRP channels, inhibit T‐type Ca^2+^ channels, and finally decrease the excitability of neurons. The adverse effects such as tolerance or respiratory depression are mediated by β‐arrestin 2, which leads to the internalization of the receptors. AC, adenylyl cyclase; cAMP, cyclic adenosine monophosphate; Ca^2+^, calcium ion; Na^+^, sodion; IRP, inwardly rectifying potassium channels; GRKs, G‐protein receptor kinases; PKC, protein kinase C

One of the leading characteristics of MORs is the biased agonism of some MOR ligands, which means that a certain ligand could induce a specific conformational change of the MORs and activate a particular signaling pathway. So far, herkinorin, oliceridine (TRV130), PZM21, mitragynine, and naltrexone (NTX)‐derived compound derivatives have been identified as the biased ligands of MORs.^109^ The analgesic and concomitant adverse effects of opioids are mainly mediated through the G‐protein pathway and β‐arrestin pathway, respectively (Figure 1).

The analgesic effects of opioids are mainly conducted by an inhibitory subunit of the G‐protein Gi. After being activated by analgesia‐biased ligands, the binding of Gi protein to MORs induces the release of the Gα subunit and Gβγ subunit complex.^110^ The α subunit of G protein inhibits the activity of adenylyl cyclase (AC), reduces the intracellular cyclic adenosine monophosphate (cAMP)‐dependent sodion (Na^+^) influx and eventually represses the excitability of neurons.^111^, ^112^, ^113^ The Gβγ subunit complex activates G‐protein inwardly rectifying potassium channels, promotes cellular hyperpolarization, and can inhibit T‐type calcium channels, decreasing calcium ion (Ca^2+^) ingress and neural depolarization.^111^, ^112^, ^113^

The adverse effects of opioids, such as tolerance and respiratory depression,^114^ are mainly mediated by the β‐arrestin pathway instead. Ligand‐activated MOR is phosphorylated by G‐protein receptor kinases or protein kinase C (PKC).^113^, ^115^ Phosphorylated MORs thus gain increased affinity to recruit and interact with β‐arrestin 2 (also known as arrestin‐3). The binding of β‐arrestin2 to MORs could uncouple or release the receptor for G‐protein signaling pathways and therefore desensitize the MORs. The MORs/β‐arrestin 2 complex then interacts with clathrin and adaptor protein 2 via the clathrin‐coated pits to endocytose and internalize in the cells.^99^, ^116^, ^117^, ^118^ Different opioids have different abilities to promote MORs internalization, with DAMGO, fentanyl, methadone, etorphine and β‐endorphin promoting robust MORs internalization, while morphine, buprenorphine (BUP) and pentazocine promoting relatively weaker internalization.^117^ In β‐arrestin knockout (KO) mice, the analgesic effect of morphine was remarkably potentiated and prolonged,^119^ and respiratory suppression and acute constipation after morphine administration were attenuated.^120^ However, although β‐arrestin KO mice did not develop antinociceptive tolerance, they still became physically dependent on morphine.^121^

## 3 REWARD CIRCUITRY, ADDICTION, AND MORS

**FIGURE 2:**
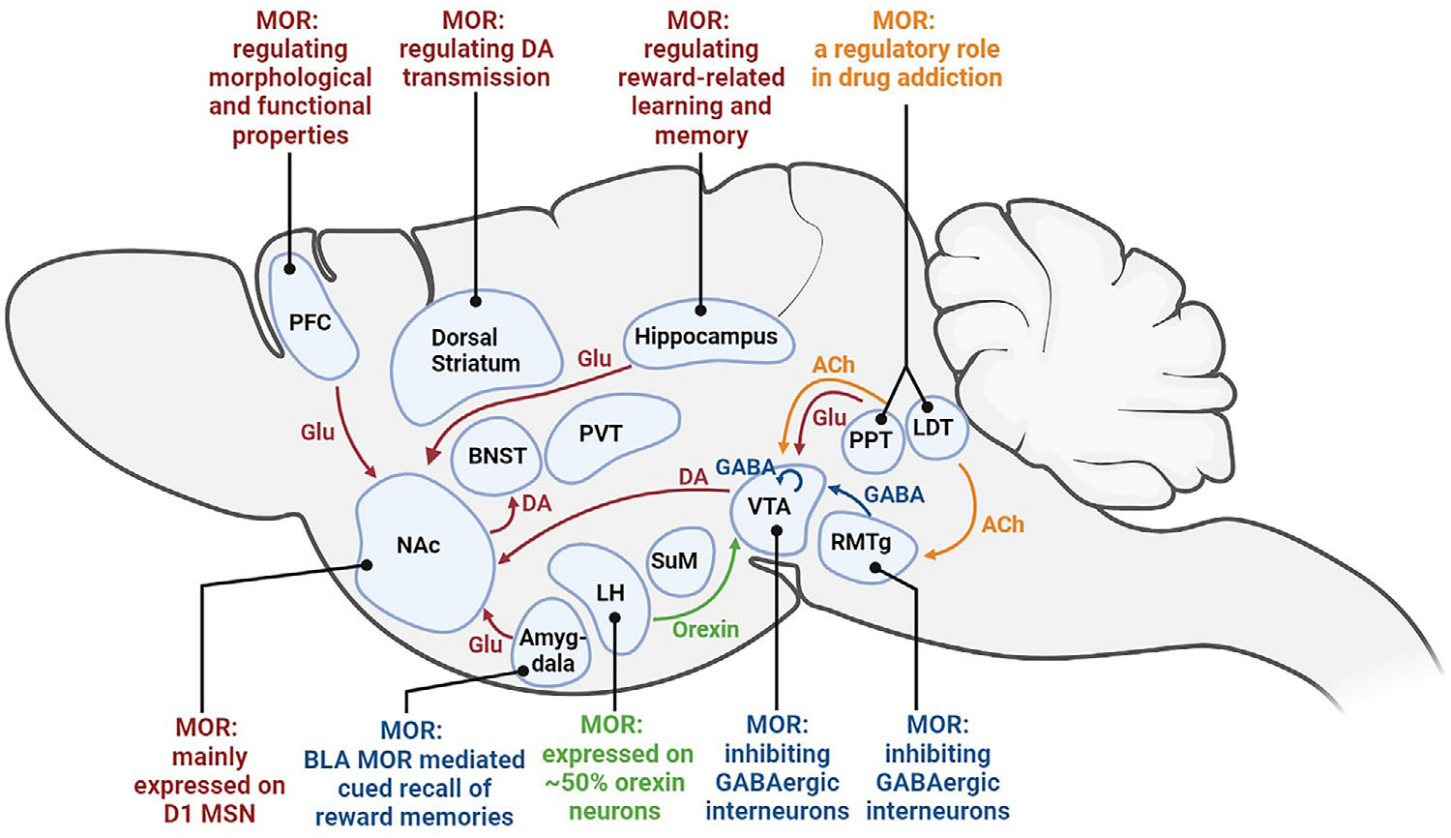
Brain regions within the reward circuitry and the role of MORs. Brain regions and nuclei that participate in reward circuitry and MORs‐mediated rewarding effects are widely distributed in the central nervous system. Ach, acetylcholine; BLA, basolateral amygdala; BNST, bed nuclei of the stria terminalis; DA, dopamine; GABA, γ‐aminobutyric acid; Glu, glutamate; LDT, laterodorsal tegmental nucleus; LH, lateral hypothalamus; NAc, nucleus accumbens; PPT, pedunculopontine tegmental nucleus; PFC, prefrontal cortex; PVT, paraventricular nucleus; RMTg, rostromedial tegmental nucleus; SuM, supramammillary nucleus; VTA, ventral tegmental area

Addiction is known as a chronic neurobehavioral disorder where addicted individuals are compulsive to seek and obtain drugs, unable to refrain themselves from taking and become dysphoric, anxious, depressed, or irritable when they do not have access to the abused drugs.^20^ Drug addiction is usually a staged process where initially the drug users have recreational or euphoric reactions to the drugs, but upon repeated consumption, they develop compulsive seeking and taking behaviors.^122^ Drugs abused often activate the brain reward circuitry, which plays a crucial role in the hedonic regulation of behaviors and initiation of addiction.^123^ The neural circuitry of the rewarding effects of abused drugs is extensively distributed in the brain, including the VTA and NAc, and the DA neurons within the VTA and NAc of the brain have been identified as the key components for the rewarding effects of abused drugs.^124^ Apart from the VTA and NAc, other brain areas such as the striatum, PFC, thalamus, hypothalamus, and amygdala are also involved in the rewarding effects of abused drugs (Figure 2).

MORs, as key modulators in the reward circuitry, play a major role in the natural reward process, regulating mood states and reward motivation.^125^, ^126^ *Oprm‐1* KO mice demonstrated deficits in social behavior and communication skills, which are deficits usually owned by drug abusers.^127^, ^128^, ^129^, ^130^ Moreover, *oprm‐1* KO mice showed a decreased motivation for food and sucrose self‐administration^131^ and failed to demonstrate an aversion to NX.^132^ Considering the wide distribution of MORs in the brain, as expected, opioids and MORs intensively regulate the reward circuitry and are thought to play a role in drug addiction. In the following section, we review the reward circuitry and brain regions that are actively involved, followed by discussing the role of MORs in reward and addiction within each brain area.

### 3.1 VTA and rostromedial tegmental nucleus (RMTg)

The VTA is a brain region that is generally thought to be the underlying pivot of drug addiction. VTA is characterized by a lack of clear boundaries and heterogeneous cellular architecture, with 60%–65% of the neuronal population being DA, 30%–35% being γ‐amino butyric acid (GABA), and 2%–3% being glutamatergic neurons.^123^, ^133^, ^134^ The activation of VTA DA neurons is the main responder to drug addiction. Tract tracing technology revealed that VTA DA neurons are composed of several subtypes with distinct projections, including projections to the NAc, the PFC, the amygdala and the hippocampus,^135^, ^136^ among which the subtype projecting to the NAc is involved in drug reward.^123^ Administration of cocaine could selectively modify the excitatory synapses of the VTA DA neurons projecting to the NAc shell by increasing the α‐amino‐3‐hydroxy‐5‐methyl‐4‐isoxazolepropionic acid (AMPA) receptor / N‐methyl‐d‐aspartate (NMDA) receptor ratios in these neurons.^137^ In addition, cocaine also inhibits the DA transporter (DAT) in the VTA, which also mediates its rewarding effect.^138^, ^139^ It remains to be elucidated which is the dominant mechanism underlying cocaine addiction, whether modifying VTA‐NAc DA projections or inhibiting VTA DAT. Anatomically, the VTA is composed of the anterior VTA and posterior VTA. It is estimated that the posterior VTA mediates drug rewarding effects more readily than the anterior zone.^124^ Apart from opioids (endomorphin‐1), drugs including nicotine, delta‐9‐tetrahydrocannabinol, and cocaine can stimulate rewards arising from the posterior VTA to activate DA neurons within the posterior VTA.^140^, ^141^, ^142^, ^143^ While VTA DA neurons are the mainstay for drug reward, it is equally clear that VTA GABAergic neurons are also critical for the reward process.^144^ The activation of VTA GABAergic neurons inhibits VTA DA neuron activity and in turn results in a decrease in DA in the NAc and disrupts reward consummatory behavior.^145^ GABAergic neurons that critically control the VTA DA are localized at the posterior tip of the VTA, called the RMTg. The RMTg is composed of a relatively pure population of GABAergic neurons, and they project to VTA DA neurons and substantia nigra pars compacta.^146^, ^147^ Stimulation of RMTg GABAergic neurons could suppress the activity of approximately 90% of DA neurons in the VTA.^148^ After reward or reward‐predictive stimuli, the activity of RMTg GABAergic neurons is inhibited.^147^

The most prevailing hypothesis for opioid reward depends on the MORs expressed in the VTA GABAergic neurons. In the VTA, MORs are mainly selectively expressed on GABAergic neurons rather than DA neurons.^149^, ^150^, ^151^, ^152^ Opioids directly act on the MORs in these GABAergic neurons, resulting in hyperpolarization and a reduced VTA GABAergic neuron firing rate and subsequently disinhibiting the activity of VTA DA neurons.^152^, ^153^ Investigations into the rewarding effects of intra‐VTA opioid administration verified the role of MORs in the VTA. Intra‐VTA microinjection of the selective MOR agonist DAMGO could induce drug‐associated conditioned place preferences in a dose‐dependent manner.^154^ In another study, bilateral microinjections of morphine into the VTA had reinforcing effects of morphine.^155^ Administration of the endogenous MOR ligand endomorphine‐1 into the VTA, especially the posterior part of the VTA, could elicit reward and increase locomotor activity.^141^ On the contrary, after NX methiodide, the MOR antagonist, was injected into the VTA, conditioned place preference induced by morphine was blocked compared with that of the control.^156^

The RMTg, or the posterior tip of the VTA, is predominantly composed of GABAergic neurons, and their major projections are the DA neurons in the VTA.^124^ The RMTg is also believed to mediate the rewarding effects of opioid administration. The RMTg GABAergic neurons were thought to provide potent inhibition to the VTA, and stimulation of RMTg GABAergic neurons could inhibit over 90% of the VTA DA neurons.^148^ Also, collected data showed that RMTg GABAergic neurons expressed high levels of MORs.^157^ After acute exposure to the psychostimulant methamphetamine or aversive stimuli of footshocks, food deprivation or reward omission, the immediate early gene *Fos* and its product FOS were detected in RMTg GABAergic neurons.^147^, ^158^ These results suggest the potential role of RMTg in reward processing and drug addiction. Another hypothesis of the MOR‐mediated reward effect arises from RMTg‐involved disinhibition of the VTA. Opioids activate MORs in the GABAergic neurons of the RMTg and inhibit the activation of these GABAergic neurons, resulting in disinhibition of VTA DA neurons and eventually an increased level of DA. Pharmacological studies have revealed the involvement of RMTg in the morphine reward process. After systemic morphine administration, the firing rate of VTA DA neurons increases through the activation of MORs in the RMTg GABAergic neurons.^159^ The inhibitory input from the RMTg controls the spontaneous firing activity of the VTA DA neurons. Electrical stimulation of the RMTg could elicit a total suppression of spontaneous activity in about half of the VTA DA neurons,^160^ and intravenous morphine could suppress the RMTg‐induced inhibition of DA neurons in vivo.^160^ Apart from morphine, the selective MOR agonist DAMGO can also decrease the spontaneous firing rate of RMTg neurons and cause hyperpolarization.^161^ Moreover, RMTg infusion of morphine and DAMGO could increase locomotor behavior in rats through MORs.^162^, ^163^ Apart from opioid‐induced reward effects, MORs in the RMTg GABAergic neurons also regulate ethanol consumption and related conditioned place preference behavior.^164^

As mentioned above, VTA DA neurons receive inhibitory inputs from both neighboring GABAergic interneurons within the VTA and GABAergic interneurons in the RMTg. Notably, after being exposed to the endogenous MOR agonist [Met5]encephalin, the input from RMTg is more strongly inhibited than those cells from the local interneurons, with GABA‐A inhibitory postsynaptic currents (IPSCs) of the former decreasing by about 75% and the latter decreasing about 17%.^165^ These results suggested that the disinhibition of VTA after morphine exposure is mostly mediated by the GABA (RMTg)‐DA (VTA) pathway rather than the local GABA (VTA)‐DA (VTA) pathway.

### 3.2 NAc

The major output regulating drug reward from the VTA DA neurons is the NAc, which is part of the ventral striatum. The medium spiny neurons (MSNs) are the subject of VTA DA projections and the predominant cellular population in the NAc. NAc MSNs belong to a heterogeneous group of GABAergic neurons that express different DA receptors, the D1 receptor or D2 receptor.^166^ D1 and D2 MSNs have different projections, with D1 MSNs being part of the “direct” pathway, which increases thalamocortical drive‐force, and D2 MSNs constituting the “indirect” pathway, which decreases thalamocortical drive.^123^, ^167^ Anatomically, the NAc can be segregated into the core, and the shell and MSNs within the different regions have different drug‐induced alterations.^168^ The MSNs in the NAc core could discriminate the motivational value of conditioned stimuli through the integration of information and synaptic plasticity at spines on the cellular surface, while MSNs in the NAc shell are involved in the behavioral consequences of repeated administration of addictive drugs.^169^ Selective stimulation of D1 receptor‐expressing MSNs of the direct pathway is sufficient to induce persistent reinforcement in both operant and place preference tasks, while activation of D2 MSNs of the indirect pathway could induce transient punishment.^170^

In the NAc, MORs are mainly expressed on D1 MSNs of the direct pathway rather than the D2 MSNs of the indirect pathway.^171^ By using a bacterial artificial chromosome‐mediated transgenic rescue strategy to re‐express MORs in D1 MSNs of the MOR KO mice, the opioid rewarding model, opiate‐induced striatal DA release, and motivation to self‐administer opiate would be restored.^171^ Moreover, morphine infused into the NAc was reported to induce and maintain self‐administration behaviors.^172^, ^173^ On the contrary, local injection of methylnaloxonium, the MORs selective antagonist, into the NAc could significantly reduce the locomotor activation effect produced by subcutaneous injection of herorin (diacetylmorphine).^174^ Other studies have interrogated the role of MORs of the NAc in the addiction other than opioid substances. MORs in the NAc shell contributes to promote binge‐like consumption of palatable foods^145^ ^,^ and they could also promote alcohol consumption, seeking and conditioned reinforcement by enhancing the incentive motivation.^175^ MORs in the NAc participate in the maintenance of local microcircuitry. A recent study demonstrated that a decrease in the copy number of MORs in the NAc resulted in increased inhibitory synaptic transmission in D2 MSN of the NAc as well as an increase in the expression of gephyrin mRNA and the density of inhibitory synaptic.^176^ Considering the crucial regulation of D2‐MSN on VTA DA neurons, such alteration caused by MORs copy number changes is supposed to be involved in reward and addiction behavior. A more recent study by Castro et al. reported that MORs regulated reward consumption behavior in mice acting through the circuit from the dorsal raphe to the NAc, and MORs‐mediated inhibition of raphe terminals is necessary and sufficient to determine the consummatory response with the source of endogenous ligands of MORs from NAc enkephalin neurons.^177^ This study revealed a novel endogenous opioid circuit that determines state‐dependent reward consumption.

### 3.3 Doral striatum

**FIGURE 3:**
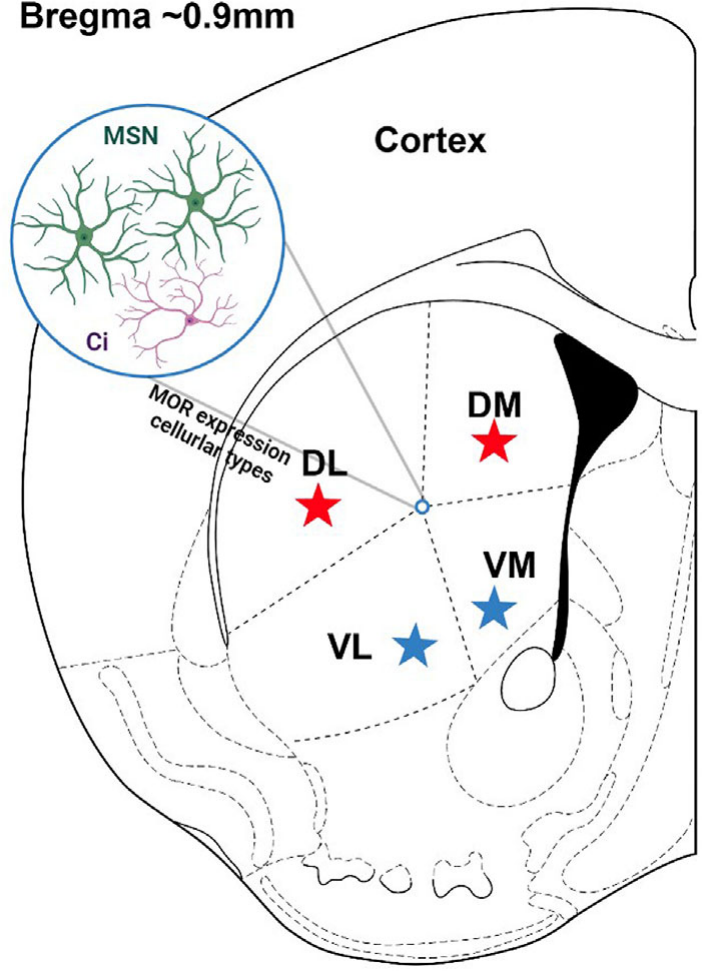
Schematic coronary view of the dorsal thalamus. The dorsal striatum is located dorsally to the nucleus accumbens and can be subdivided into four territories according to the spatial distribution. The four territories of the dorsal striatum include the DL striatum, the DM striatum, the VL striatum, and the VM striatum. MORs are highly expressed on MSNs and Ci in the dorsal striatum. The DL striatum and DM striatum were reported to be involved in the drug addiction process (denoted as red star), while little attention has been given to the role of the VL and VM striatum (denoted as blue star) in drug addiction. Ci, cholinergic interneuron; DL, dorsolateral; DM, dorsomedial; MSN, medium spiny neuron;; VL, ventrolateral; VM, ventromedial

The brain striatal complex can be anatomically divided into the dorsal striatum and the NAc, and the former can further be separated into four sub‐territories with different neurochemical and neuroanatomical properties, the DL, the dorsomedial (DM), the ventrolateral (VL), and the VM^178^, ^179^ (Figure 3). The corticostriatal network is believed to control heterogeneous decision‐making processes, including both goal‐directed and stimulus bound, highly involved in reward and drug addiction.^180^, ^181^, ^182^ The DL striatum goes through drastic neurochemical and functional changes during drug addiction. In rats that were trained to self‐administer cocaine, glutamate signaling increased in the DL striatum, and antagonism of AMPA receptor increased the efficacy of cue extinction to reduce drug craving.^183^ Meanwhile, in rats tolerant to ethanol and nicotine, long‐term depression was occluded in the glutamatergic synapses in the DL striatum, suggesting the contribution of drug addiction to the alteration of synaptic plasticity.^184^ The involvement of the DL striatum in stimulus‐response learning was further evidenced by a study where ablation of the neurons of the patch compartment of the DL striatum by dermorphin‐saporin resulted in reduced reinstatement of sucrose self‐administration after sucrose devaluation.^185^ Interestingly, the involvement of the DL striatum in drug abuse seems to be influenced by gender as is suggested by the activation of G protein‐coupled estradiol receptor 1 in the DL striatum that could enhance motivation for cocaine and drug‐induced reinstatement in females rather than male rats.^186^, ^187^ The involvement of the DM striatum has been implicated by the fact that animals with DM striatum excitotoxic lesions selectively affected the behavioral adjustment to a situation involving reward uncertainty,^188^ and repeated nicotine administration altered the local field potential in the DM striatum.^189^ More recent works revealed the differential involvement of the MSNs of the direct (dMSNs) and indirect pathways (iMSNs) of the DM striatum. In a probabilistic Pavlovian conditioning task, dMSNs were involved in suppressing ongoing licking behavior, while iMSNs contributed to outcome‐dependent behavioral adjustment.^190^ More importantly, another study found that optogenetic stimulation of the DM striatum‐external globus pallidus (GPe) iMSNs reduced ethanol‐containing reward‐seeking, whereas optogenetic inhibition of the DM striatum‐GPe iMSNs reversed this change.^191^ The activity of MSNs in the DM striatum and related reward behavior is regulated by local astrocytes, at least in part. Kang et al. reported that activation of DM striatum astrocytes decreased the spontaneous excitatory postsynaptic currents (sEPSCs) in dMSNs while increasing sEPSCs in iMSNs and facilitated shifting from habitual to goal‐directed reward‐seeking behavior.^192^ Little attention has been given to the regulatory role of the VM and VL striatum in reward and addiction. One possible explanation might be that the VM and VL striatum are in the proximity of the NAc, and the majority of studies focused on the NAc rather than the VM and VL striatum. In fact, generally, the NAc is referred to as the “ventral striatum” in the literature.^193^, ^194^, ^195^ Further studies are needed to elucidate the role of VM and the VL striatum in the control of reward and addiction.

The involvement of dorsal striatum MORs in the regulation of reward and addiction was unraveled by early studies showing that opiate administration could increase the DA concentration in the dorsal caudate nucleus in rats.^196^ Further studies demonstrated that in the dorsal striatum, presynaptic activation of MORs reduced glutamate and GABA release, postsynaptic activation of MORs reduced DA release, and MORs mediated the long‐term depression of excitatory inputs to the dorsal striatum.^197^, ^198^, ^199^, ^200^ Interestingly, MORs‐dependent modulation of basal DA transmission in the rat dorsal striatum was reported to be region‐specific as evidenced by the observation that DAMGO (an MORs selective agonist) in the rostral and caudal dorsal striatum reduced DA levels, while it increased DA levels in the medial dorsal striatum.^201^ Moreover, prolonged treatment with morphine led to reduced dendritic arborization and loss of dendritic spines in the MSNs of the dorsal striatum, which was mediated by D4 receptors on MSNs.^202^ In spite of the aforementioned studies, there are currently few studies directly investigating the causal relationship between MORs and drug abuse behaviors. Further studies that directly regulate MORs in different cellular and structural compartments of the dorsal striatum by genetic manipulation methods or optogenetics might provide more knowledge.

### 3.4 PFC

The NAc not only receives DA inputs from the VTA but also receives glutamatergic inputs from the PFC, the thalamus, the ventral hippocampus, and the amygdala.^203^, ^204^ Cellular adaptions in the glutamatergic projection from the PFC to the NAc have been discovered in rats withdrawn from cocaine; that is, the altered G protein signaling in the PFC underlies the behavior on drug‐related stimuli, while dysregulated PFC‐NAc synaptic glutamatergic transmission could be the reason for the unmanageable drug‐seeking.^205^ The PFC also has a regional preference in its projection to the NAc. The infralimbic (IL) medial PFC (mPFC) largely projects to the shell of the NAc, and the prelimbic (PrL) mPFC prefers the core of the NAc.^204^ Optogenetic activation of the IL‐mPFC‐NAc and PrL‐mPFC‐NAc exerts different effects with the former potentiating and the latter inhibiting cocaine craving behavior, respectively.^206^ In addition, another study reported that optogenetic stimulation of the PFC‐NAc pathway promoted conditioned reward‐seeking behavior after learning, while activity in activation of the PFC‐paraventricular nucleus (PVT) of the thalamus suppressed both the acquisition and expression of conditioned reward‐seeking.^207^ Recently, by means of single‐cell RNA sequencing, a broad impact of cocaine on transcription was observed across the PFC. Especially during the withdrawal phase, the transcriptional impact is extremely prominent.^208^

Opioid receptors in the PFC have unneglectable modulatory effects on both the morphological and functional properties of the local network, which subsequently interferes with the output signal of the PFC and thus influences the reward circuitry. A postmortem study found that in opioid drug abusers, the expression of the GluN1 and GluN2B but not the GluN2A subunits of the NMDA receptors was increased in both the mPFC and the lateral PFC.^209^ Moreover, further animal studies demonstrated that chronic morphine administration could significantly increase the total dendrite length and dendritic complexity of both PV interneurons and somatostatin interneurons in the mPFC.^210^ Another study showed that chronic cocaine administration increased the level of MORs mRNA in the PFC.^211^ As for the functional regulatory role of MORs in the PFC, Witkowski et al. found that the activation of MORs expressed in the non‐pyramidal neurons of the mPFC inhibited the voltage‐dependent Na^+^ currents in a protein kinase A‐ and PKC‐dependent manner,^212^ while Olianas discovered that concomitant activation of DORs and MORs in the mPFC potentiated DA D1‐like receptor signaling.^213^ DA signaling is indispensable for MORs to exert their modulatory roles. Infusions of DAMGO into the VM PFC could induce augmented sucrose‐reinforced responding, while blocking the D1 receptors of the VM PFC simultaneously attenuated such effect, suggesting that D1 tone plays an enabling or permissive role in the expression of MORs‐elicited effects.^214^ This study also demonstrated that simultaneous targeting of the MORs and the DA system might be a more efficacious strategy to counter addiction characterized by dysregulated appetitive motivation.

### 3.5 Thalamus

Inputs from subregions of the thalamus to the NAc have also been characterized to regulate the drug rewarding process. The PVT and the supramammillary nucleus (SuM) are two widely investigated regions. The PVT is a part of the midline and intralaminar thalamic group and is an interface for brain reward circuits.^215^ Previous studies demonstrated that the activation of the PVT is also associated with a predisposition to reinstate to cocaine‐seeking elicited by drug‐related cues,^216^ while inactivation of the PVT could prevent the conditioned place preference induced by cocaine.^217^ A further two‐photon calcium imaging study by Otis et al. found that PVT neurons projecting to the NAc developed inhibitory responses to reward‐predictive cues coding for both cue‐reward associative information and behavior that were directed by the activity of prefrontal and lateral hypothalamic afferent axons,^218^ further confirming the relaying characteristics of PVT‐NAc projection in the reward circuits. The input from the PVT to the NAc mediates the expression of opioid‐withdrawal‐induced physical signs and aversive memory, and activation of this input pathway is sufficient and necessary to mediate behavioral aversion.^219^ Chronic morphine exposure could selectively potentiate the excitatory transmission between the PVT and the D2 MSNs in the NAc.^219^

The SuM is localized in the posterior hypothalamic and is thought to participate in the drug reward process.^124^ The SuM can mediate reward triggered by the GABA_A_ receptor antagonist picrotoxin, nicotine, and the glutamate receptor agonist AMPA.^143^, ^220^, ^221^ The SuM is also reported to interact with the VTA‐NAc system in reward. SuM injections of AMPA were reported to increase extracellular DA in the NAc measured by microdialysis.^220^ In addition, intra‐VTA injections of the cholinergic agonist carbachol, which is reported to induce reward effects such as conditioned place preference and vigorous locomotion, could induce significant c‐Fos expression in the SuM, and such an increase in the c‐Fos is positively correlated with vigorous locomotion induced by the intra‐VTA injections of carbachol.^222^ These findings suggest the role of SuM in the reward process, but its detailed participation needs further articulation. Although the mRNA of MORs was detected in the SuM,^223^ the particular role of SuM MORs in reward and drug addiction remains to be understood in future work.

### 3.6 Hypothalamus

The hypothalamus, especially the lateral hypothalamus (LH), has also been reported to be involved in drug addiction and reward as is directly evidenced by the observation that deep brain stimulation of the LH reduced cocaine‐seeking behavior.^224^ The hypothalamus, including the LH, is enriched in neuropeptides that have important roles in thermoregulation, sleep, feeding, sex drive, and general motivational behavior.^225^ As for the role in drug addiction, the most widely recognized LH neuropeptide is orexin, also named hypocretin. Orexins, including orexin A and orexin B, were identified as endogenous ligands for GPCRs of two orexin receptors, OxR1 and OxR2.^226^, ^227^ Orexins are widely recognized for their crucial regulatory roles in feeding behavior and circadian rhythm as evidenced by the reports that intracerebroventricular injection of orexins induced feeding in rodents, and orexin deficiency caused narcolepsy in humans and animals.^228^, ^229^, ^230^ In addition to feeding and circadian rhythm, orexin neurons in LH are important players in reward processing and drug abuse.^231^ Early studies demonstrated that rewarding stimulus would activate orexin neurons in the LH. Harris et al. reported that the activation of LH orexin neurons was linked to preferences for cues associated with drug and food reward, and chemical activation of these groups of neurons reinstated an extinguished drug‐seeking behavior that was completely blocked by prior administration of orexin antagonist.^232^ In another report, consistently, chronic exposure to amphetamine for 5 days also activated LH orexin neurons.^225^ Moreover, the rewarding‐behavior corresponding activation of orexin neurons was reported to be exclusively located in the LH rather than orexin neurons outside the LH such as in the perifornical or DM hypothalamus, or non‐orexin neurons in the LH.^225^ Neuroanatomically, the control of LH orexin neurons over drug addiction is mostly determined by its connectivity with the VTA. Projections from the LH to the VTA affect both DA neurons and GABAergic interneurons in the VTA, the two critical types of neurons in the VTA that regulate reward and addiction as previously mentioned. Borgland et al. reported that orexin A induced potentiation of NMDA‐mediated neurotransmission in VTA DA neuron synapses in vitro and that in vivo administration of an OxR1 antagonist occluded cocaine‐induced potentiation of excitatory currents in VTA DA neurons, suggesting the role of orexin signaling in neural plasticity of VTA DA neurons.^233^ In another report, stimulation of LH orexin neurons mainly inhibited VTA DA neurons and activated GABAergic neurons, suggesting that the effect of orexin on VTA DA neurons was strongly mediated by local interneurons.^225^

Around 50% of LH orexin neurons express MORs.^234^ Early studies in which morphine was injected into the LH enhanced local c‐Fos expression and food consumption confirmed the role of MORs in the regulation of the function of LH.^235^ Further study investigating the gene expression profile of LH indicated that chronic morphine exposure significantly altered the gene expression of LH in morphine‐dependent mice, suggesting that LH MORs might contribute to the morphine addictive response.^236^ Moreover, upon morphine withdrawal after chronic exposure, the mRNA levels of both MORs and orexin increased.^234^ MORs in the LH regulate feeding behavior through orexin neurons;^237^ however, the exact relationship and mechanism of MORs and orexin in the regulation of addiction‐related behavior are not clear.

### 3.7 Amygdala and the extended amygdala (EA)

The amygdala also sends glutamatergic projections to the NA, and its effects are thought to be mediated by specific DA receptors, with D1 agonists but not D2 agonists attenuating amygdaloid inputs. The basolateral amygdala (BLA), which has a crucial role in emotional learning, is critical for the reward modulation. The BLA neuronal response to rewarding cues precedes the reaction of NAc neurons, and cue‐evoked excitation of NAc neurons depends on BLA input.^238^ In mice, optogenetic stimulation of the pathway from the BLA to the NAc could reinforce behavioral self‐stimulation of these synaptic inputs, and this effect relies on NAc D1 receptors rather than D2 receptors.^239^ On the other hand, optogenetic inhibition of the BLA‐NAc pathway could reduce cue‐evoked intake of sucrose.^239^ In drug addiction, incentive motivation often becomes narrowly focused on the particular drug of abuse. Central nucleus of amygdala (CeA) activation could facilitate this kind of narrowing of motivation. When rats are provided the option for intravenous cocaine exposure, optogenetic stimulation of the CeA could intensify that option to become the exclusive focus of pursuit and consumption.^240^ The centromedial nucleus of the amygdala (CeMA) also has a role in reward‐related behaviors. Optogenetic activation of GABAergic projection from the CeMA to the VM PFC could produce a positive reward‐like phenotype in real‐time place preference and increase locomotor activity and nose‐poking effort in sucrose operant conditioning.^241^

**FIGURE 4:**
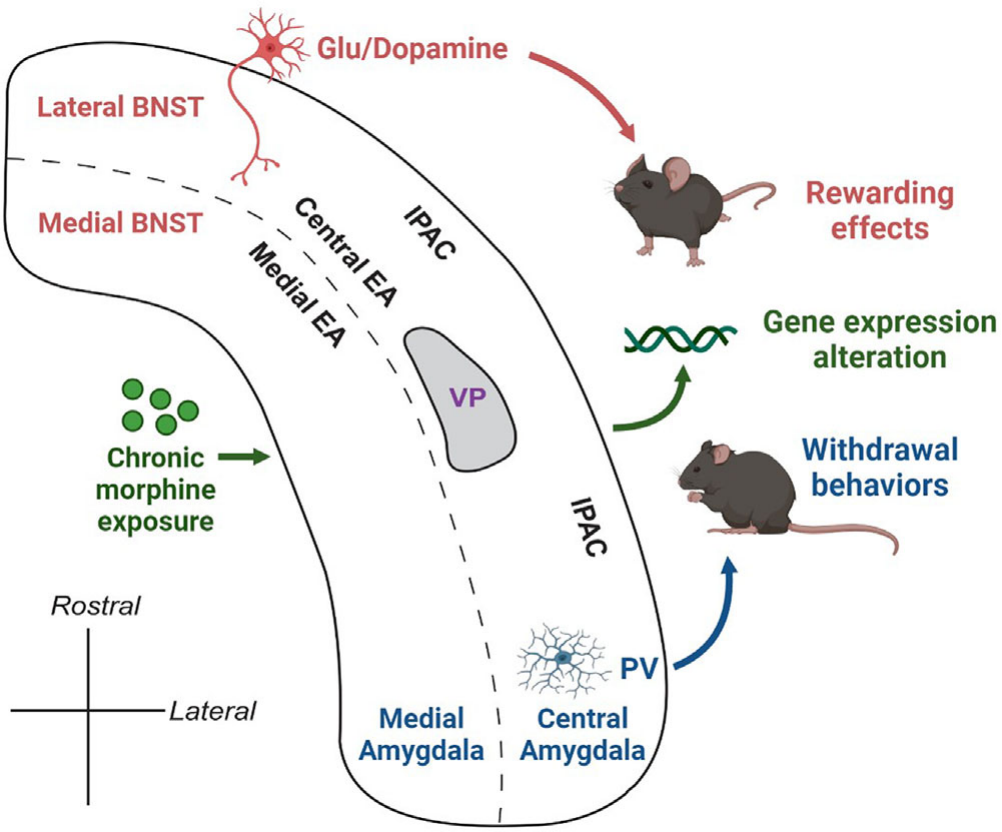
Schematic horizontal view of the extended amygdala (EA) continuum showing the composition of brain regions. The central EA extends from the central nucleus of the amygdala through IPAC to the lateral bed nuclei of the stria terminalis (BNST). Central EA surrounds the VP. The medial EA is located medially to the central EA and contains the medial nucleus of the amygdala and the lateral BNST. Glu and dopaminergic neurotransmission in the BNST is involved in the rewarding aspects of drug addiction, while PV interneurons are involved in withdrawal behaviors of dependent subjects. Chronic morphine exposure could significantly alter the gene expression profiles of EA. Glu, Glutamatergic; IPAC, interstitial nucleus of the posterior limb of the anterior commissure; PV, parvalbumin; VP, ventral pallidum

The EA is a basal forebrain macrostructure situated between the amygdala and the striatopalidum, running from the dorsal amygdala through the substantia innominate to the bed nuclei of the stria terminalis (BNST) and the NAc shell^242^, ^243^ (Figure 4). The EA can be separated into the central EA and the medial EA, with the former part containing the CeA and the lateral portions of the BNST and the medial EA containing the medial nucleus of the amygdala and medial BNST.^244^ It remains controversial whether the shell of the NAc is part of the EA.^245^, ^246^ EA is an important player in drug addiction as well as reward circuitry. EA is highly involved in the control of aversive processes, such as fear responses, stress, anxiety, and particularly opioid withdrawal effects.^247^, ^248^ It has been demonstrated that the application of NX to the CeA could elicit withdrawal jumping behavior in morphine‐dependent rats, while bilateral electrolytic lesion of the CeA eliminated the withdrawal jumping behavior.^249^ Notably, chronic morphine withdrawal increased the firing rate of the PV interneurons in the CeA, while optogenetic inhibition of the activity of CeA PV interneurons attenuated the morphine withdrawal‐induced negative affective states, such as aversive, anxiety, and anhedonic‐like behaviors.^250^ This study suggested that the MORs on the PV interneuron might play a major role in CeA‐regulated withdrawal behavior. In addition to the control of aversive situations, EA has also been suggested in reward learning and opioid addiction. When food reward or cocaine was earned by operant responding, the excitatory glutamatergic neurotransmission increased in the ventro‐lateral BNST,^251^ suggesting the involvement of the BNST after an appetitive stimulus. The BNST is heavily innervated by mesolimbic DA neurons originating in the VTA, and morphine, nicotine, cocaine, and ethanol could significantly increase the extracellular DA in the BNST, suggesting the sensitivity of the BNST to the DA stimulant actions of drugs of abuse.^252^ Blocking the DA D‐1 receptor in the BNST decreased the cocaine reinforcement effect.^253^ Chronic activation of MORs in the central EA led to dysregulation of genes clustered into neurogenesis, cell growth, and signaling proteins, suggesting that MORs in the central EA contributed to drug‐induced neural plasticity.^254^ Accordingly, microinjections of the opiate receptor antagonist methylnaloxonium into the BNST suppressed heroin self‐administration in dependent rats, further confirming the role of EA MORs in drug addiction.

Evidence exists that MORs in the nuclei of the amygdala and EA contribute to the regulation of local activity and reward‐related behaviors. Prenatal morphine exposure in male rats reduced and increased the density of MORs in the BLA and CeA, respectively, without influencing the MORs level in the BNST.^255^ In the BLA, MORs are involved in the encoding of incentive value, which underlies the development of the desire for morphine continuous intake.^256^ Another study demonstrated that BLA MORs mediated the cued recall of reward memories, allowing rats to motivate action.^257^ Evidence indicates that opioid abuse is associated with NMDA receptor‐dependent plasticity in the CeA and BNST. Beckerman et al. found that GluN1 and MORs were co‐expressed in CeA‐BNST projecting neurons and in the axonal terminals in the BNST, and further deletion of GluN1 in CeA neurons resulted in the decrease in morphine‐induced fos expression in the ventral BNST.^258^ This study suggested that NMDA receptors are essential for MORs‐mediated activity in the BNST and opioid addictive behaviors. Moreover, GluR2‐expressing non‐calcium‐permeable AMPA receptors were also reported to be colocalized with MORs in the CeA.^259^ However, its implication for reward behaviors remains to be elucidated. MORs activation significantly altered the gene expression profiles in EA, where samples of CeA and BNST were pooled together,^254^ where chronic morphine exposure, instead of a single morphine injection, induced overrepresentation of genes governing neurogenesis, cell growth and signaling protein categories from gene ontology analysis.^254^

### 3.8 Hippocampus

The hippocampus is also involved in reward behavior. A population of cells associated with reward has been identified in the hippocampus, and hippocampal activity could be altered under contextual rewarding stimuli.^260^, ^261^, ^262^ The hippocampus also has glutamatergic projections to the NAc, and this kind of innervation is critical for the reward response. Induction of long‐term potential at the synapses of the hippocampus‐NAc inputs could drive conditioned place preference, and the activity of these synapses is required for the response to natural reward.^263^ The ventral hippocampus is involved in associative memory and emotional behavior, which are associated with morphine exposure. The morphine‐induced conditioned place preference could significantly facilitate neurogenesis in the ventral dentate gyrus (DG) and increase the dendritic spine density in both the CA1 and DG,^264^ suggesting that morphine‐induced reward memory is related to neural and synaptic plasticity in the ventral hippocampus.

The hippocampus is the hub of memory.^265^ Reward‐related learning and memory contribute to compulsive drug use and addiction.^266^ It was reported that MORs agonists could significantly increase the amplitude of sharp waves and the occurrence of sharp‐wave ripples (a specific electrophysiological activity pattern of the hippocampus underlying the consolidation of memory), as well as increase the network excitability of CA1 regions, suggesting that MORs in the CA1 might contribute to addiction by enhancing drug memory consolidation.^267^ Moreover, the adult hippocampus is an active site of neurogenesis where neural stem cells in the DG undergo proliferation and differentiation.^268^ Zhang et al. demonstrated that morphine self‐administration, a paradigm mimicking human opiate addiction, increased neural stem cells differentiation and dendritic growth in the adult DG, which is mediated by MORs expressed on neural stem cells. Moreover, they found that conditional overexpression of MORs in DG neural stem cells led to enhanced morphine self‐administration, confirming the role of DG MORs in the establishment of morphine addiction.^269^ Furthermore, another two studies reported that cocaine addiction increased the MORs expression and functionality in the rat hippocampus,^270^ and adolescent morphine exposure enhanced the MORs‐mediated G‐protein activity in the hippocampus and morphine preference in the conditioned place preference test in adult mice.^271^ These two studies provide us with a glimpse of hippocampal MORs alteration under drug addiction situations.

### 3.9 Laterodorsal tegmental (LDT) nucleus and pedunculopontine tegmental (PPT) nucleus

The LDT nucleus and the PPT nucleus send projections to the VTA, and they are also found to be involved in drug rewarding behavior.^272^, ^273^ Both the LDT and PPT have heterogeneous cellular subpopulations of cholinergic (acetyltransferase, [ChAT]), GABAergic (glutamic acid decarboxylase [GAD]) and glutamatergic (vesicular glutamate transporters 2) cells.^274^ Electrical stimulation of the LDT and PPT could evoke DA increase in the NAc and striatum, respectively, with the former relying on the nicotinic and glutamatergic receptors in the VTA and the latter relying on the nicotinic and glutamatergic receptors in the substantia nigra.^275^, ^276^ Optogenetic studies have revealed the role of LDT and PPT in rewarding behavior. The LDT preferentially sends synapses onto VTA DA neurons projecting to the NAc lateral shell, and optogenetic stimulation of the LDT‐VTA inputs could elicit reward such as strong conditioned place preference.^277^ Furthermore, optogenetic intracranial self‐stimulation of the LDT‐VTA inputs could increase DA in the NAc, and this increase depends on NAc D1 and D2 receptors.^278^ On the other hand, in rats, selective optogenetic stimulation of PPT cholinergic inputs to the VTA could result in positive reinforcement.^279^ Optogenetic activation of glutamatergic PPT projection to the VTA could preferentially excite VTA DA neurons, and this is sufficient to induce behavioral reinforcement.^280^ These results suggest the potential role of LDT and PPT in drug addiction.

Studies regarding MORs in the LDT and PPT and the relevance with reward and addiction are limited. However, the existing studies demonstrate that MORs in LDT and PPT play a regulatory role in drug addiction. An early study by Klitenick et al. reported that bilateral microinjections of DAMGO into the PPT elicited a dose‐dependent increase in motor activity and also an increase in extracellular DA content in the NAc,^281^ a core brain region involved in the reward circuitry as previously described. However, this study does not deal with drug addiction behavior. Further study conducted by Corrigall et al. provided direct evidence that infusion of DAMGO into the PPT produced a dose‐related reduction in the number of cocaine self‐administration, suggesting that the MORs in the PPT could influence drug‐reinforced behavior.^282^ The influence of PPT or LDT MORs on drug addiction might be mediated by their projection to RMTg. RMTg was reported to receive inputs from LDT and PPT.^146^ Wasserman et al. further demonstrated that LDT and PPT cholinergic neurons project to RMTg, and such cholinergic inhibition of RMTg GABA neurons via M4 muscarinic receptors facilitates the opioid inhibition on the same neuron.^157^ Future studies are needed to focus on the direct functional influence of MORs on LDT and PPT local circuitry and its relevance to drug addiction.

## 4 MORS AND OPIOID WITHDRAWAL SYNDROME (OWS)

### 4.1 OWS

As previously described, MORs play a crucial regulatory role in the reward circuitry through which opioids exert their rewarding and hedonic effects. The rewarding and hedonic effects of drug addiction belong to one important aspect, which initiates and urges drug users to continuously pursue the euphoria provided by the drugs. Noticeably, another indispensable aspect of opioid addiction is the OWS, which usually leads to the failure of the attempt to get rid of the use of opioids and reinforces addiction behavior.^283^ In fact, in addition to rewarding effects, negative reinforcement is a recognized model that composes addiction, pointing out that escape or avoidance of negative affect is the principal motive for addictive drug use.^284^ In the following part of this review, we will continue to discuss the role of MORs in OWS, followed by introducing current strategies to treat opioid addiction and dependence by targeting MORs.

The severity of OWS varies among different patients depending on the type of the abused opioid, the duration of use, the medication history and family history.^285^, ^286^ There is a time course of OWS after opioid discontinuation, with the severity of OWS symptoms peaking during the early phase of discontinuation and gradually tails off into the late phase. Abrupt discontinuation from short‐acting opioids such as heroin and oxycodone is responsible for intense OWS that usually begins within 12 h after opioid cessation, peaks at 36–72 h and then gradually calms down during the following 4–7 days. On the contrary, abrupt withdrawal from long‐lasting opioid, such as BUP, usually demonstrates less severe OWS than short‐acting opioids, but the OWS could last for 2 weeks or even more.^27^ However, regardless of the categories of the abused opioid, the OWS during the acute phase of discontinuation is usually difficult for the patient to bear, and without proper treatments, many patients are ultimately unable to endure the withdrawal process, resulting in opioid relapse.^287^, ^288^ Acute withdrawal syndrome is usually composed of symptoms including aches, muscle spasms, abdominal cramps, nausea/vomiting/diarrhea, irritability, insomnia, tachycardia, lacrimation, sweating, and rhinorrhea.^285^, ^289^ Moreover, emotional deficits such as despair, anxiety, and anhedonia could also develop after opioid withdrawal.^290^, ^291^ In patients who initially took opioid because of chronic pain, 56.5% of them chose to continuously use opioid in order to avoid withdrawal symptoms.^292^ In addition, after short‐term medically supervised withdrawal from opioids without long‐term medication assistance, the relapse rate is as high as 77%,^293^ and this is usually associated with death resulting from drug overdose.^294^, ^295^ Thus, withdrawal symptoms often lead to the failure of opioid discontinuation, reinforce opioid addiction and bring about serious consequences to the abusers.

### 4.2 The role of MORs in opioid withdrawal

**FIGURE 5:**
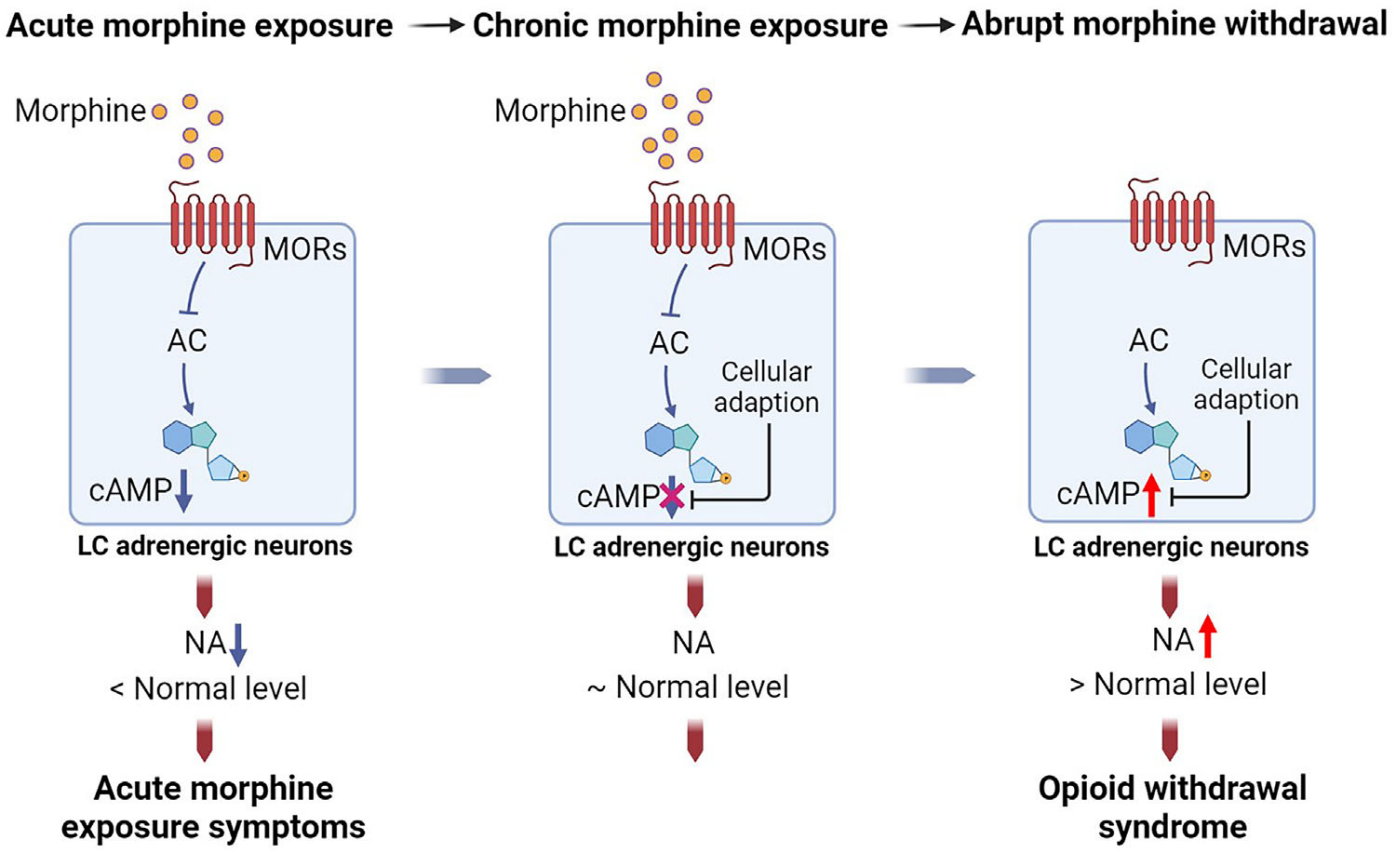
The role of MORs in the development of opioid withdrawal syndrome. MORs are highly expressed in adrenergic neurons in the locus coeruleus (LC). During acute morphine exposure, the activation of MORs by morphine inhibits the activity of adenylyl cyclase (AC), which leads to a decrease in cAMP and subsequent norepinephrine (NA) release. When morphine is chronically administered, adaptation of the LC adrenergic neurons results in the normalization of intracellular cAMP levels. When the supply of morphine stops, the inhibitory effects of morphine on AC diminish, leading to the excess production of cAMP and release of NA, which triggers the occurrence of morphine withdrawal syndrome, including symptoms of aches, muscle spasms, anxiety, and so forth

The prolonged exposure to opioid results in multiple adaptive changes in the CNS. The OWS and related dependence on opioid is mainly attributed to alterations in the locus coeruleus (LC), which is mediated by the chronic activation of the MORs (Figure 5). The majority of neurons in the LC are adrenergic neurons or neurons containing norepinephrine (NA), projecting to various regions of the brain, including the PFC, the hippocampus and the amygdala.^296^ Under physiological conditions, the LC adrenergic neurons projections can stimulate wakefulness, regulate breath and blood pressure, and maintain alertness.^297^, ^298^, ^299^ MORs are abundant in LC adrenergic neurons. When opioids bind to and activate LC adrenergic MORs, they can inhibit the activity of adenylyl cyclase (AC), thus suppressg the production of cAMP, leading to decreased release of NA eventually. The decreased NA innervation could result in acute opioid effects, such as drowsiness, reduced respiration and blood pressure, and decreased muscle tone.^27^, ^299^ Upon chronically repeated doses of opioids, adaptions of the LC neurons occur, and the level of cAMP gradually returns to a normal level before opioid exposure; thus, the LC neurons then release the normal amount of NA. However, when opioids are no longer supplied and the inhibitory effect of repeated opioids on LC NA neurons is eliminated, the neurons would produce excessive amount of cAMP, and NA would be excessively released, triggering aches, muscle spasms, abdominal cramps, anxiety, and so forth, the symptoms of OWS.^27^, ^299^

As previously mentioned, after being activated by opioids, the binding of Gi protein to MORs induces the release of α subunit, and the α subunit inhibits the activity of AC and thus reduces the intracellular levels of cAMP.^110^, ^111^, ^112^, ^113^ Sustained activation of LC NA neurons would uncouple MORs from the Gi protein α subunit, resulting in the reduction of the inhibitory effect on AC activity and the re‐upregulation of the AC/cAMP pathway.^300^, ^301^ The MORs located on the lipid raft are necessary for the superactivation of AC after chronic opioid exposure. After long‐term MOR agonists (morphine, etorphine, and methadone) treatment, the majority of MORs remain on the lipid raft; the treatment of methyl‐beta‐cyclodextrin, a raft‐disrupting agent, could completely blunt the AC superactivation.^302^ Apart from the abovementioned Giα uncoupling mechanism, AC superactivation is also mediated by Src kinase. Chronic activation of MORs could further recruit Src kinase to phosphorylate MOR at Tyr336 within the NPXXY motif, and the lack of such Src kinase‐mediated phosphorylation of MORs results in complete blunting of AC activation.^303^, ^304^

MORs in other regions are also reported to be involved in the generation of withdrawal syndrome. MORs in the dorsal raphe nucleus area (DRN) contribute to the depressive symptoms after opioid abstinence. Genetic KO of MORs in the serotonergic neurons in the DRN before opioid exposure could abolish the development of social withdrawal after opioid withdrawal.^305^ This result implies that MORs could regulate serotonergic transmission in the DRN and MORs alteration after chronic opioid exposure may contribute to the psychiatric symptoms during opioid withdrawal. Another study observed that during the extended withdrawal period of 10 days, the expression of MORs in the caudate‐putamen, frontal, and cingulate cortices was increased.^306^ After a withdrawal period of 31 days, there was a decrease in the MORs protein level in the striatum, and this was considered to serve as a substrate for relapse to drug‐seeking.^307^

## 5 CURRENT STRATEGIES TO TREAT OPIOID ADDICTION AND DEPENDENCE VIA MORs

Considering the high prevalence of opioid abuse, the intractable withdrawal and dependence situation of opioid abstinence, and the crucial role of MORs in opioid addiction, it is of great importance to treat opioid addiction focusing on MORs with agents with long‐lasting efficiency. The medication treatment of opioid addiction contributes to the prevention of relapse, which can help the addicted individuals to be stable enough to return to work and normal social interaction with periods of abstinence as long as possible.^308^, ^309^ The MORs are the main targets of treatment of addiction. As previously discussed, both the analgesic and adverse effects of opioids are mediated by MORs. However, currently, the efforts to design an agent that can exert analgesia without the likelihood of being abused have been unsuccessful.^310^ The available treatment options for opioid addiction now are mainly focused on the prevention of the development of dependence, the elimination of dependence, and the suppression of withdrawal symptoms.^19^, ^299^, ^311^

**FIGURE 6:**
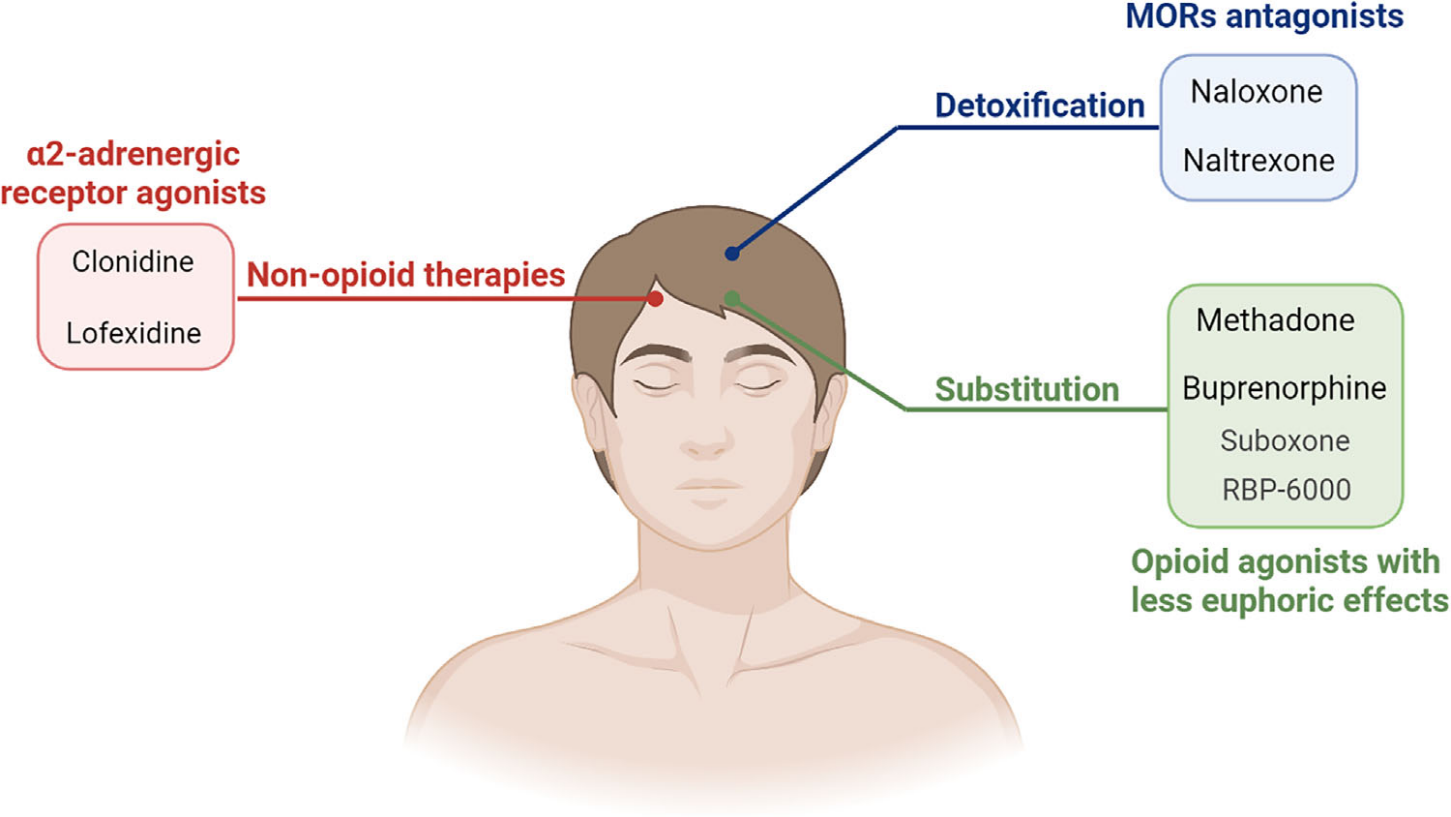
Strategies to treat opioid addiction and dependence. Current strategies to treat opioid addiction mainly involve detoxification therapy followed by maintenance of opioid substitution therapy. Detoxification therapies harness MOR antagonists such as naloxone and naltrexone to reverse the acute intoxication effects. Substitution therapies include dose‐monitored opioid agonists, methadone and buprenorphine (with formulations of Suboxone and RBP‐6000), with lasting and less euphoric effects to reduce withdrawal syndrome. α2‐Adrenergic receptor agonists, including clonidine and lofexidine, are non‐opioid therapies targeting the withdrawal symptoms caused by norepinephrine hyperactivity during opioid abstinence

Current pharmacological strategies to tackle opioid addiction and dependence can mainly be classified into the following categories: (1) detoxification therapy using opioid antagonists (NTX and NX); (2) opioid substitution therapy using longer‐acting MORs agonists with less euphoric effects (methadone and BUP, etc.); (3) non‐opioid therapies, that is, α2‐adrenergic receptor agonists clonidine and lofexidine^299^, ^309^, ^312^ (Figure 6). However, medications for opioid use disorders are underused. First, only a small percentage of patients who survived opioid overdose received medications for opioid use disorder.^313^ In addition, when medications are prescribed, BUP and methadone are frequently given with insufficient dose and/or for too short duration.^19^ Clinical trials on agents to treat opioid use disorders/dependence are summarized in Table 1.

**TABLE 1:** Clinical trials on agents to treat opioid use disorder/opioid withdrawal syndrome

**Agents**	**Category**	**Interventions**	**Main results**	**Ref**.
Naloxone (NX)	Mu opioid receptors (MORs) antagonist	Orally taken NX	Orally taken NX improved symptoms of opioid associated constipation and reduced laxative use	^343^, ^344^
		Intravenously taken NX	Intravenous NX reversed the morphine‐induced respiratory depression in healthy volunteers	^345^
		Sustained‐release NX capsule, orally taken	Successful treatment of opioid‐induced constipation without comprising the desired opioid effects	^346^
Naltrexone (NTX)	MORs antagonist	Extended‐release (XR) NTX, intramuscular injection	XR NTX was associated with a lower rate of opioid relapse than usual treatment	^347^
		Injectable XR NTX	Injectable XR NTX significantly increased opioid‐free days and decreased craving for opioids in patients with opioid dependence disorder	^348^
		Injectable XR NTX versus oral NTX	Injectable XR NTX was associated with twice the rate of treatment retention at 6 months, compared with oral NTX	^349^
Methadone	MORs agonist	Daily oral methadone hydrochloride	Both moderate‐ and high‐dose methadone resulted in decreased illicit opioid use. The high‐dose group had significantly greater decreases in illicit opioid use	^350^, ^351^
		Methadone maintenance therapy	Methadone maintenance therapy was efficacious in reducing heroin use and human immunodeficiency virus (HIV) risk behaviors	^352^
		High‐dose and low‐dose methadone	Compared with low‐dose methadone, levomethadyl acetate, buprenorphine (BUP), and high‐dose methadone substantially reduced the use of illicit opioids	^353^
BUP	MORs agonist	BUP, administered three times a week	BUP substantially reduced the use of illicit opioids, compared with low‐dose methadone	^353^
		Sublingual tablets consisting of BUP and NX or BUP alone	BUP and NX in combination and BUP alone were safe and reduced the use of opiates and the craving for opiates among opiate‐addicted patients	^354^
		Continuing treatment was 24‐mg BUP‐NX per day for 9 weeks and then tapered to week 12 versus short‐term detoxification was 14‐mg BUP‐NX per day and then tapered to Day 14	Continuing treatment with BUP‐NX improved the outcome, compared with short‐term detoxification in opioid‐addicted youth	^355^
		Sublingual BUP‐NX tablets, followed by four BUP implants	Compared with placebo, BUP implants resulted in less opioid use over 16 weeks among persons with opioid dependence	^356^
		Sublingual BUP‐NX tablets, followed by four BUP implants	BUP implants did not result in an inferior likelihood of remaining a responder	^357^
		BUP taper versus ongoing BUP maintenance therapy	Tapering was less efficacious than ongoing maintenance treatment in patients with prescription opioid dependence who received BUP therapy in primary care	^358^
		A novel, weekly, subcutaneous BUP depot formulation, CAM2038	CAM2038 was safely tolerated and produced immediate and sustained opioid blockade and withdrawal suppression	^359^
		Daily BUP hydrochloride with NX hydrochloride versus XR NTX	XR NTX was as effective as BUP‐NX in maintaining short‐term abstinence from heroin	^360^
		Intramuscular XR‐NTX versus daily self‐administered BUP‐NX sublingual film	It was more difficult to initiate patients to XR‐NTX than BUP‐NX, which negatively affected overall relapse. Once initiated, both medications were equally safe and effective	^361^
		Monthly subcutaneous injection of BUP‐XR	Participants' percentage abstinence was significantly higher in BUP‐XR groups than in the placebo group. Treatment with BUP‐XR was also well‐tolerated	^338^
		BUP‐NX versus XR‐NTX	BUP‐NX was more cost‐effective than XR NTX to prevent opioid relapse in patients with opioid use disorder	^362^
Clonidine	α2‐adrenergic receptor agonist	Clonidine, orally taken	Clonidine was efficacious to block acute opiate‐withdrawal symptoms	^340^
		Clonidine versus methadone detoxification	Clonidine may not be superior to methadone in terms of the number of patients able to achieve abstinence	^363^
		Opioid‐dependent patients who maintained abstinence during Weeks 5 and 6 were continued on BUP and randomly assigned to receive clonidine or placebo for 14 weeks	Clonidine was an adjunctive maintenance treatment that increased the duration of abstinence	^364^
Lofexidine	α2‐adrenergic receptor agonist	BUP detoxification versus lofexidine detoxification	BUP is at least as effective as lofexidine opiate detoxification treatment	^365^
		Lofexidine–NTX versus placebo–NTX	Patients with lofexidine–NTX had higher opioid abstinence rates and improved relapse outcomes as well as attenuated stress and drug cue‐induced opioid craving response	^366^
		Lofexidine and clonidine were each tested as pretreatments once in combination with intramuscular NX	Neither lofexidine nor clonidine suppressed the subjective discomfort of opioid withdrawal or significantly reduced other autonomic signs of opioid withdrawal	^367^

Typical opioid use disorder treatment involves detoxification therapy followed by maintenance of opioid substitution therapy, with the former aiming at the reversal of the intoxication caused by opioid overuse and the latter aiming at progressive reduction in OWS and relapse.^310^, ^314^ The detoxification therapy involves opioid antagonists. NX is a potent non‐selective MORs competitive antagonist that can reverse the acute intoxicating effects of opioid overdose such as respiratory depression.^315^ NX can exert its effects rapidly. Intramuscular or intravenous administration of NX could restore respiratory depression within 1 to 2 min.^310^ Apart from intramuscular or intravenous administration, intranasal NX was reported to be as effective as intravenous NX in reversing both the depressive effects on the CNS and respiratory depression caused by opioid overuse.^316^ Intranasal NX provides a convenient approach to administer, which could be extremely useful in some emergency situations. NTX is another MORs antagonist. Studies have found that extended‐release (XR) NTX is effective in maintaining short‐term withdrawal from heroin,^317^, ^318^ and its economic costs are also acceptable.^319^

Opioid substitution therapy involves dose‐controlled, long‐acting opioid agonists with less euphoric effects, which could reduce withdrawal syndrome and inhibit craving for opioids. Methadone and BUP are the two most commonly used agents in opioid substitution therapy. Methadone is a full MORs agonist and remains the gold standard in the treatment of opioid addiction.^320^ It has a long half‐life with an average around 22 h,^321^ and its efficacy varies with different doses. Lower dosages (20–40 mg/day) of methadone are sufficient to suppress the emergence of opioid withdrawal symptoms, but it may not be enough to block the craving for opioids.^310^, ^320^ It was demonstrated that a daily dosage ranging from 60 to 100 mg is more effective than a lower dosage in reducing the use of heroin and cocaine during the treatment.^322^ In addition to the decrease in opioid use, in a 17‐year longitudinal cohort study, methadone maintenance therapy is also associated with lower rates of offending crime.^323^

BUP is a partial MORs agonist with a high affinity for MORs,^324^ while it is also an invert agonist at KORs and an antagonist at DORs.^325^ In addition, BUP also acts as a full agonist at a fourth category of receptors, the opioid receptor‐like 1 (ORL1, also known as NOP).^326^ The interaction of BUP with MORs is characterized by four aspects, including (1) low efficacy or partial agonism, meaning that the maximal effect of BUP is less than that of a full MORs agonist, (2) high affinity, meaning that BUP is difficult to displace from the MORs, (3) high potency, meaning that low doses of BUP might be enough to elicit the same degree of effects by high doses of other agonists, and (4) slow dissociation, meaning that BUP has a long duration of action.^327^ Because of its nature as a partial MOR agonist and slow dissociation, BUP is associated with less sedation and euphoria effects than methadone, and it also has long‐term action to treat withdrawal symptoms and decrease mortality.^328^ Moreover, BUP does not activate biased signaling of MORs, avoiding the activation of the β‐arrestin pathway and thus diminishing the adverse effects of MORs activation when morphine was co‐administered with BUP.^325^, ^329^ Notably, BUP also activates ORL1, which complicates its action through MORs. OLR1 has high sequence similarity with other opioid receptors and is coupled with similar second messengers.^330^ Lutfy et al. reported that the concomitant activation of OLR1 compromised the MOR‐mediated effects of BUP.^331^ This was evidenced by their finding that in ORL1 KO mice, the antinociceptive effect of BUP was markedly enhanced.^332^ They further demonstrated that activation of ORL1 by BUP also compromised the rewarding effects of BUP as evidenced by BUP behaving as a full MORs agonist and inducing greater rewarding effects in mice lacking ORL1.^333^ Compared with the lower dose of BUP (less than 16 mg/day), a higher dose of BUP (16–32 mg/day) is more efficient to achieve better retention in treatment as suggested by a meta‐analysis.^334^ This dose‐dependent effect of BUP could involve its role at ORL1 as suggested by the fact that BUP concentration‐dependently displaced the specific binding of nociception, the endogenous ligand of ORL1.^333^ The regulatory importance of ORL1 on the activity of MORs encouraged the development of ORL1/MORs (also called NOP/MOP) bifunctional agonists in order to achieve analgesia without causing addiction and abuse.^335^ Fortunately, efforts have been conducted to develop and test such agents. Early investigation illustrated that SR16435, an ORL1/MORs bifunctional agonist, was more potent than morphine in attenuating pain with slower development of tolerance in mice.^336^ Recent advances also provided preclinical evidence that AT‐121, which had partial agonist activity at both ORL1 and MORs, exerted morphine‐like analgesia without causing side effects such as respiratory depression, abuse potential, and physical dependence.^337^ Currently, the most frequently used formulation of BUP is named Suboxone (BUP:NX, 4:1), which has very low potential to be abused.^299^, ^310^ Recently, a monthly administered, XR BUP therapy, referred to as RBP‐6000 or BUP‐XR, was found to be applicable to achieve abstinence and was also well‐tolerated.^338^ This monthly formulation represents an advance in the treatment of opioid abuse that both enhances the benefits of BUP and reduces the risks of BUP.

The α2‐adrenergic receptor agonists are the representatives of non‐opioid therapies, and they can target the withdrawal symptoms caused by NA hyperactivity during opioid abstinence through autoreceptor feedback inhibition.^339^ Clonidine and lofexidine are the two mainstay α2‐adrenergic receptor agonists used to treat opioid addiction, and both have shown efficacy in treating opioid withdrawal.^340^, ^341^ The efficacy difference is not detected between clonidine and lofexidine, but clonidine is associated with unwanted hypotension than lofexidine more frequently.^342^

## 6 CONCLUSION AND FUTURE PERSPECTIVES

The dominant roles of opioids in analgesia and their strong euphoric temptation determine that opioid abuse and addiction would still be a prevalent problem in the future. MORs play a protagonist part in the reward system regulation and dependence development caused by long‐term adaption, regardless of these effects being wanted or unwanted. MORs also bear the significance of being the target of relieving opioid addiction and dependence. With a more elaborate understanding of the circuitry contributions and signal transduction characteristics of MORs, more effective drugs will be developed to overcome opioids abuse. A possible option is to prepare a monoclonal antibody that targets MORs and stabilizes MORs in a certain conformation. However, concerns of the mode of administration and related adverse effects still exist. In addition, the development of drug screening would help us find more analgesics with mild or even no risks of being addicted. In conclusion, MORs are central to opioid addiction; at the same time, they present us with a possible gate to treat drug abuse and dependence in the future.

## CONFLICT OF INTEREST

The authors declare that there is no conflict of interest.

## AUTHOR CONTRIBUTIONS

Z.J.J. and S.C.G. searched and screened the literature, wrote the draft, and drew the figures and tables. J.M.D. and L.L. assisted in the search of literature, the drawing of figures, and the critical revision of the article. Y.X.M. and Z.N.C. critically revised the article and figures, supervised the coordination and were in charge of correspondence. Z.J.J. and S.C.G. contributed equally to this article. All the authors have agreed to the final submitted version of this article.

## ETHICS STATEMENT

Not applicable.

## Data Availability

Data sharing is not applicable to this review, as no new data were created or analyzed.
